# Influence of feedforward control-based health education intervention on compliance, visual function and self-perceived burden among patients with diabetic retinopathy

**DOI:** 10.4314/ahs.v23i3.39

**Published:** 2023-09

**Authors:** Jia Wang, Huanyu Tang

**Affiliations:** Affiliated Nanhua Hospital, University of South China, Hengyang 421001, Hunan Province, China

**Keywords:** Correlation, diabetic retinopathy, feedforward control, health education, self-perceived burden, visual function

## Abstract

**Objective:**

To assess the influence of feedforward control-based health education intervention on the compliance, visual function and self-perceived burden (SPB) among patients with diabetic retinopathy (DR).

**Methods:**

Eighty-six DR patients were divided into feedforward control and control groups (n=43). The control group was given routine nursing intervention, based on which the feedforward control group received feedforward control-based health education intervention. The health behavior indices were compared after intervention. The correlations of QOL score with SPB score and health behavior indices were analysed using Pearson's and Spearman's coefficients.

**Results:**

After intervention, the total QOL score and scores of symptoms and visual function, physical function, social activity, and mentality and psychology were significantly improved compared with those before intervention, which were significantly higher in the feedforward control group (P<0.05). SPB score was significantly lower in the two groups after intervention than that before intervention, particularly in the feedforward control group (P<0.05). The QOL score of DR patients was significantly negatively correlated with SPB score but positively correlated with health behavior indices (P<0.05).

**Conclusion:**

The feedforward control-based health education intervention mode is beneficial for guiding DR patients to promote visual function recovery and to reduce SPB.

## Introduction

Diabetes mellitus (DM) is a clinically common chronic disease. The number of DM patients is increasing annually in China, also endangering the younger population^1^. Diabetic retinopathy (DR), as one of the severe ocular complications in DM patients, is clinically manifested as the continuous elevation of blood glucose level and enhancement of blood coagulation, leading to microvascular damage, local thrombosis, vascular breakdown, diminution of vision and even blindness in severe cases^2^,^3^. At present, DR can be classified into proliferative and non-proliferative types. It is mostly treated with drug intervention or surgery, but the risk of blindness remains high. Until now, the effective methods for DR prevention and treatment remain lacking, so the quality of life (QOL) of patients is greatly threatened^4^. Therefore, the clinical control of DR is not only limited to the blood glucose control and reduction of incidence rate of complications through hypoglycemic drugs. Instead, it is also necessary to guide patients to maintain scientific and reasonable daily habits and to establish correct self-management awareness^5^.

Feedforward control is an intervention model based on a comprehensive understanding of patients' basic information, early education about possible risks, prediction of adverse outcomes in advance, and formulation of targeted measures, aiming to improve the awareness of prevention and to decrease the risks as much as possible^6^.

On the above basis, a feedforward control-based health education intervention plan was designed, and its influence on the compliance, visual function and self-perceived burden (SPB) of DR patients was evaluated in this study, aiming to provide a potentially effective regimen for ameliorating the prognosis of DR patients.

## Materials and methods

### General data

A total of 86 DR patients treated in our hospital from August 2021 to January 2022 were selected and divided into feedforward control group (n=43) and control group (n=43) by stratified random sampling. In the feedforward control group, there were 25 males and 18 females aged 45-68 years old, with an average of (56.17±4.39) years old. The duration of disease was 1-8 years, with an average of (4.13±1.05) years. Type 1 diabetes mellitus (T1DM) and T2DM occurred in 9 and 34 patients, respectively. There were 31 patients with non-proliferative DR (NPDR), including 20 type I cases, 7 type II cases and 5 type III cases, and 12 patients with type IV proliferative DR (PDR). In the control group, there were 23 males and 20 females aged 42-66 years old, with an average of (55.92±4.15) years old. The duration of disease was 1.5-9.5 years, with an average of (4.26±1.12) years. T1DM and T2DM occurred in 11 and 32 patients, respectively. There were 34 patients with NPDR, including 21 type I cases, 6 type II cases and 7 type III cases, and 9 patients with type IV PDR. The baseline data, including gender ratio, age, duration of disease, stage of DM and stage of retinopathy, had no significant differences between the two groups (P>0.05).

The inclusion criteria were as follows: (1) patients meeting the diagnostic criteria for DR according to clinical manifestations and imaging results7, (2) those with complete clinical data, and 3) those who had no history of mental illness or manifestation of mental disturbance, had a clear consciousness, and agreed to participate and cooperated in this study till the end. The exclusion criteria were as follows: (1) patients complicated with other macular degeneration diseases, (2) those who had undergone the treatment of macular edema before admission, including vitreous surgery or glucocorticoid therapy, (3) those complicated with vision diseases such as high myopia, hyperopia or astigmatism, (4) those with vascular disease around the retina, or (5) those complicated with severe organic diseases in the liver or kidney.

### Intervention methods

The patients in the control group were given routine nursing intervention as follows. The blood glucose level and blood pressure of patients were measured daily, and medication and dietary guidance was given based on their vital signs and clinical symptoms. On the above basis, the patients in the feedforward control group received feedforward control-based health education intervention as follows.

(1) A group was established based on the health education mode of feedforward control, including 1 head nurse of the department as the leader and 6 nurses as the group members. With this intervention mode, the investigator was responsible for training all members regarding the concept and specific steps of the nursing plan, and the guidance and relevant methods of medication, dietary intake and limb exercise, emphasizing the particularity of nursing DR patients and the risk factors in the nursing process, developing effective measures, and regularly assessing the group members.

(2) Summary of experience: A meeting was held to search for cases, and to summarize the compliance, treatment methods, risk factors, visual function, SPB, basic procedures and precautions of DR patients. The group members communicated with each other. The nursing intervention strategies for possible risks were formulated to prepare the final plan. In clinical application, the plan was gradually improved to ensure high-quality implementation.

(3) Health education was given to all patients, and the possible risk factors for the progression of disease, including fasting and 2-hour postprandial blood glucose level, body weight, blood pressure and blood lipid level, were monitored. The monitoring results and condition of disease were diagnosed and assessed by experienced chief physicians, and the targeted nursing intervention plan was developed according to the patients' actual situation.

(4) The clinical senior nurses explained the causes, clinical manifestations and influencing factors of DM and DR and the precautions during nursing, and popularized the corresponding nursing knowledge according to the visual function, so the patients better understood the disease, and their clinical cooperation and enthusiasm for self-management were enhanced.

(5) Dietary guidance: The clinical nurses developed a scientific and reasonable dietary intake plan according to the individual situation of patients, and explained to them the importance of dietary intake control for visual functional recovery, thereby improving the clinical compliance of patients and standardizing the behavior of following doctor's instruction. Protein intake of >15%, fat of <30%, carbohydrate intake of 55-65% and cholesterol intake of <300 mg/d should be ensured in the dietary guidance plan.

(6) Limb exercise intervention: The nurses collected the clinical data of each patient, clarified their baseline data and condition of disease, effectively assessed the daily habits and disease history, developed practical and effective exercise plan combined with the patients' actual situation, guided them to implement the plan, and helped them maintain normal metabolism and body weight.

(7) Drug intake guidance: The patients were guided to use drugs as the doctor instructed. The high-risk patients were subjected to blood glucose monitoring on time, accompanied by insulin intake to control the blood glucose level. Attention should be paid to the clinical manifestations of patients in real time, and any abnormality should be reported to the doctor promptly. Non-high-risk patients should be instructed to establish a scientific and reasonable life consciousness and to standardize their living habits, and guided to maintain the correct method of insulin injection. However, the possible side effects of insulin injection should be noticed, and the senior nurses should try to avoid using long-acting insulin.

(8) The group members paid attention to the emotional and psychological states of DR patients in time, enhanced the communication, and provided professional and targeted psychological counselling based on the emotional expression of patients, thereby helping them keep a positive attitude in treatment and build confidence in overcoming the disease.

(9) Out-of-hospital guidance should be given to patients, and return visit should be made according to the patients' actual situation, generally once a month to determine the progression of disease.

### Detection of biochemical indices

Before and after intervention, 8 mL of fasting venous blood was drawn from each patient in the morning and separated by VARIANTII high-performance liquid chromatography (Bio-Rad, USA). Then the biochemical indices fasting plasma glucose (FPG), glycosylated hemoglobin (HbA1c), triacylglycerol (TG), high-density lipoprotein (HDL) and low-density lipoprotein (LDL) levels were detected using 7080 automatic biochemical analyzer (Hitachi, Japan).

### Observation indices

(1) The differences in the levels of various biochemical indices, including FPG, HbA1c, TG, HDL and LDL, were compared between the two groups. (2) The differences in the health behaviors, including medication compliance, blood glucose self-monitoring rate, completion rate of return visit, and awareness rate of health care knowledge, were compared between the two groups. (3) The changes in QOL score were compared between the two groups before and after intervention, including the total QOL score and the scores of symptoms and visual function, physical function, social activity, and mentality and psychology. (4) The self-perceived burden scale (SPBS) was used to compare SPB between the two groups before and after intervention. (5) The correlations of the QOL score of DR patients with SPB score and health behavior indices were analysed using Pearson's and Spearman's coefficients.

### Assessment criteria for health behaviors

The patients were followed up through telephone call or WeChat to assess their health behaviors using a selfmade health behavior questionnaire 1 month after discharge, including medication compliance, on-time return visit, blood glucose self-monitoring and awareness rate of health care knowledge. The score of each item ranged from 1 to 10 points, and ≥9 points indicated behavioral health. The Cronbach's coefficient alpha of the questionnaire was 0.823, and the test-retest validity was 0.815^8^.

### Assessment criteria for QOL

QOL of DR patients was assessed using the Scale of Quality of Life for Eye Diseases with Visual Impairment^9^, including 20 items in 4 dimensions (social activities, physical function, clinical symptoms and visual function, and mentality and psychology). A higher score meant higher QOL of patients.

### Assessment criteria for SPB

SPBS consists of 10 items, each ranging from 1 to 5 points, “1”: never, “2”: occasionally, “3”: sometimes, “4”: often, and “5”: always. Except item 8, all items are forward integration. The sum of points of all items was the total score of SPBS, and a higher score meant higher burden. The total scores of <20 points, 20-30 points, 30-40 points and ≥40 points indicated no SPB, mild SPB, moderate SPB and severe SPB, respectively^10^.

### Statistical analysis

SPSS 26.0 software was used for statistical analysis. The normally distributed measurement data were expressed as mean ± standard deviation (x ± s), and subjected to the t-test between two groups and compared by the paired t-test within the same group. The count data were expressed as (n, %), and subjected to the χ^2^ test between African Health Sciences, Vol 23 Issue 3, September, 2023 two groups. The correlations of the QOL score of DR patients with SPB score and health behavior indices were analysed using Pearson's and Spearman's coefficients. P<0.05 was considered statistically significant.

## Results

### Changes in the levels of biochemical indices before and after intervention

Before intervention, no significant differences were found in the biochemical indices including FPG, HbA1c, TG, HDL and LDL between the two groups (P>0.05). After intervention, biochemical indices significantly declined in the two groups compared with those before intervention, and they were significantly lower in the feedforward control group than those in the control group (P<0.05) (Table 1).

**Table 1:** Changes in the levels of biochemical indices before and after intervention (mmol/L, ^-^x ± s)

Group	FPG		HbA1c		TG		HDL		LDL	

	Before intervention	After intervention	Before intervention	After intervention	Before intervention	After intervention	Before intervention	After intervention	Before intervention	After intervention
Feedforward control (n=43)	7.25±0.96	6.21±0.79*	9.64±1.11	8.36±0.85*	3.62±0.58	2.65±0.41*	1.76±0.34	1.18±0.22*	5.12±0.60	3.98±0.46*
Control (n=43)	7.14±0.88	6.83±0.84*	9.55±1.06	9.03±0.97*	3.51±0.52	3.13±0.47**	1.69±0.31	1.42±0.27*	4.98±0.54	4.51±0.49*
*t*	0.554	-3.526	0.385	-3.407	0.926	-5.047	0.998	-4.519	1.137	-5.171
P	0.581	0.001	0.702	0.001	0.357	<0.001	0.321	<0.001	0.259	<0.001

* P<0.05 *vs.* before intervention.

### Changes in health behaviors

Compared with the control group, the medication compliance, completion rate of return visit, blood glucose self-monitoring rate, and awareness rate of health care knowledge were significantly improved in the feedforward control group (P<0.05) (Table 2).

**Table 2:** Changes in health behaviors (n, %)

Group	Medication compliance	Completion rate of return visit	Blood glucose self-monitoring rate	Awareness rate of health care knowledge
Feedforward control (n=43)	36(83.72)	38(88.37)	37(86.05)	39(90.70)
Control (n=43)	24(55.81)	27(62.79)	25(58.14)	26(60.47)
χ^2^	7.938	7.623	8.323	10.648
P	0.005	0.006	0.004	0.001

### Changes in QOL score of patients before and after intervention

Before intervention, there were no significant differences in the scores of symptoms and visual function, physical function, social activity, and mentality and psychology, and total QOL score between the two groups (P>0.05). After intervention, theses indices significantly rose in the two groups compared with those before intervention, and they were significantly higher in the feedforward control group than those in the control group (P<0.05) (Table 3).

**Table 3:** Changes in QOL score of patients before and after intervention (point, x̅ ± s)

Group	Symptom and visual function		Physical function		Social activity		Mentality and psychology		Total QOL score	

	Before intervention	After intervention	Before intervention	After intervention	Before intervention	After intervention	Before intervention	After intervention	Before intervention	After intervention
Feedforward control (n=43)	38.04±6.36	47.62±7.43	11.75+3.83	21.57±4.82*	10.43±2.82	15.94±3.25*	12.79±2.46	18.27±3.49*	73.01±14.87	103.40±18.13*
Control (n=43)	37.25±5.94	41.17±6.72*	12.11+4.02	15.16±4.39*	11.01±3.05	12.87±3.12*	13.06±2.58	14.83±3.24*	73.43±15.43	84.03±16.36*
*t*	0.595	4.222	-0.425	6.447	-0.916	4.468	-0.497	4.737	-0.129	5.201
P	0.553	<0.001	0.672	<0.001	0.362	<0.001	0.621	<0.001	0.898	<0.001

* P<0.05 *vs.* before intervention.

### SPB score before and after intervention

There was no significant difference in the SPB score between the two groups before nursing intervention (P>0.05). The SPB score was significantly lower in the two groups after intervention than that before intervention, particularly in the feedforward control group (P<0.05) (Table 4).

**Table 4:** SPB score before and after intervention (point, x̅ ± s)

Group	Before intervention	After intervention
Feedforward control (n=43)	35.17±6.83	20.16±4.74*
Control (n=43)	34.85±6.51	27.39±5.42*
*t*	0.222	-6.885
P	0.825	<0.001

* P<0.05 *vs.* before intervention.

### Correlations of QOL score with SPB score and health behavior of DR patients

**Figure 1:**
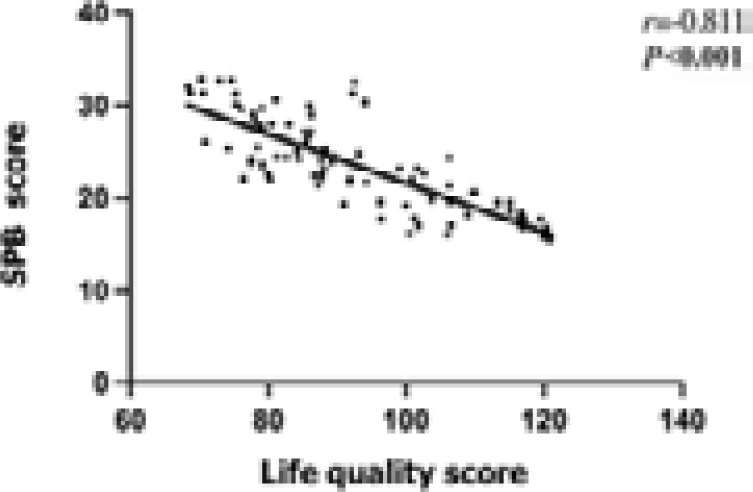
Correlations of QOL score with SPB score

According to the Pearson's and Spearman's coefficients, the QOL score of DR patients was significantly negatively correlated with SPB score but positively correlated with health behavior indices (P<0.05) (Table 5 and Fig. 1).

**Table 5:** Correlations of QOL score with health behavior of DR patients

Index	QOL score	

	*rs*	*P*
Medication compliance	0.735	<0.001
Completion rate of return visit	0.667	<0.001
Blood glucose self-monitoring rate	0.737	<0.001
Awareness rate of health care knowledge	0.701	<0.001

## Discussion

Most DR patients have insufficient awareness of their own disease, and fail to deeply understand relevant health care knowledge, master knowledge of scientific nursing, maintain scientific and reasonable dietary and exercise habits according to their own circumstances, and establish the awareness of regular fundus examination, so they rarely see doctors in the case of mild diminution of vision, thus increasing the incidence rate of DR^11^,^12^. With the continuous development and updating of nursing intervention modes in the medical field, the modern bio-psychological-social medical nursing concept has been established and improved. The feedforward control-based health intervention mode is mainly to transmit and popularize health care knowledge and effective nursing concepts through various media, and to guide patients to set up a healthy and scientific lifestyle while gaining an in-depth understanding of disease control knowledge, so the prognosis and QOL of DR patients can be improved^13^,^14^.

After examination on admission, some DM patients learn from doctors that a long-term diabetic state can lead to eye diseases, but they never receive eye examinations, thus losing the opportunity of early treatment15. In this study, the blood glucose and blood lipid levels of DR patients were effectively controlled in the feedforward control group and better improved than the control group, and the health behaviors of the feedforward control group were significantly superior to those of the control group, similar to the findings of Keel *et al.*^16^ The above results may be related to the nursing intervention plan. The feedforward control-based health intervention mode is mainly to provide scientific and reasonable nursing plans about medication, dietary intake and limb exercise based on the progression of disease and patients' physical condition, and to conduct effective health education and psychological counselling in time according to patients' emotional and psychological states, so the patients can change their daily habits and establish correct belief and confidence in treatment. In addition, the QOL score and SPB score were compared between the two groups in this study. The feedforward control group had a significantly higher QOL score than that of the control group, being consistent with the results of Ting *et al.*^17^ Possibly, the feedforward control-based<=“” span=“” style=“-font-family: “Times New Roman”;”>increased the patients' cognition of their own disease and awareness rate of relevant health care knowledge, allowed them to better recognize the health belief, and helped them build a mindset to actively overcome the disease while improving their compliance, as the driving force for behavior changes. Moreover, the knowledge and preventive measures of DR were explained to patients in this study. As a result, the patients had a clearer understanding of the progression and prognosis of disease, and regularly received examination and treatment^18^,^19^.

Notably, the psychological state and emotion of DR patients have close associations with the maintenance of subsequent health behaviors. Negative emotions affect not only the compliance of patients, but also the improvement of visual function. Therefore, how to effectively relieve the negative emotions of DR patients is one of the major concerns of clinical staff^20^. We herein found that both nursing methods relieved the negative emotions of these patients, but the feedforward control-based intervention was more effective. Likewise, DR patients often have negative emotions mainly due to the long duration of disease, anxiety of the treatment effect and poor cognition of health knowledge^21^. With the feedforward control-based health education intervention mode, the psychological problems of patients can be analysed, which is highly important for alleviating their negative psychological state. Meanwhile, the compliance of DR patients during diagnosis and treatment can be significantly improved, and the visual function is also better recovered. Besides, in this study, both SPB score and health behaviors were closely related to the QOL score, indicating that maintaining health behaviors effectively controlled biochemical indices such as blood glucose and blood lipid levels, and developed good daily habits, which played an important role in controlling the progression of disease. From another point of view, a decreased SPB score indicates that the patient can face the disease with a positive and optimistic attitude, actively cooperate in treatment and receive rehabilitation training, thereby enhancing the therapeutic effect and ameliorating the prognosis^22^. In clinical nursing, therefore, we should pay attention to the emotional state of DR patients, and guide them to better cooperate with medical staff during rehabilitation training with a positive attitude and to achieve out-of-hospital self-management, thereby improving their QOL.

Regardless, this study still has limitations such as the short follow-up period and small sample size, which may cause deviations in the results. In the future, the sample size and range will be expanded, and the follow-up period will be extended to provide references for subsequent development and improvement of corresponding clinical nursing intervention plans.

In conclusion, the feedforward control-based health intervention mode can effectively control the blood lipid and blood glucose levels of DR patients, enhance the visual functional recovery, standardize the health behaviors, and also help them develop good daily habits. Through popularizing disease-related health knowledge and conducting effective psychological counseling, this intervention mode has positive significance for raising the patients' enthusiasm for treatment, and can also ameliorate their QOL. Hence, this mode is worthy of clinical popularization and application.
